# Reduced need for inpatient care following introduction of long-acting injectable buprenorphine

**DOI:** 10.1186/s12913-025-13607-7

**Published:** 2025-10-22

**Authors:** Emelie Gauffin, Antonio Marques Franca, Elena Pizzaro Ferrero, Zeb Freij, Isa Pihlflyckt, Mikael Sandell, Charlotte Gedeon, He Zhang, David Andersson, Gustav Tinghög, Andrea Johansson Capusan

**Affiliations:** 1 Center for Social and Affective Neuroscience, Department of Biomedical and Clinical Sciences, Linköping University, Linköping, S-581 85 Sweden; 2 Department of Psychiatry in Linköping, and Department of Biomedical and Clinical Sciences, Linköping University, Linköping, Sweden; 3 Beroendekliniken, Sahlgrenska University Hospital, Gothenburg, Sweden; 4 Regional Clinic of Psychiatry, Östersund and Psychiatric clinic of Region Västernorrland, Östersund, Sweden; 5 PRIMA Maria Opioid Agonist Therapy Clinic, Stockholm, Sweden; 6 Solstenen Addiction Centre, Lund, Sweden; 7 Linköping University, Forum Östergötland, Linköping, Sweden; 8 Department of Management and Engineering, Division of Economics, Linköping University, Linköping, Sweden; 9 Department of Health, Medicine and Caring Sciences, The National Center for Priority Setting in Health Care, Linköping University, Linköping, Sweden

**Keywords:** Opioid use disorder, Opioid agonist treatment, Long-acting injectable buprenorphine, Health care utilization

## Abstract

**Background:**

Growing evidence supports long-acting injectable (LAI) buprenorphine in opioid use disorder treatment. Implementation is limited due to concerns about costs as well as potential reduction of support, which could increase strain on health care services. This real-life study follows clinical patients one year prospectively and one year retrospectively from switching from daily sublingual or peroral opioid agonist treatment (OAT) to LAI buprenorphine exploring how this impacts days of inpatient care and emergency room (ER) visits.

**Methods:**

From electronic medical records, from four Swedish OAT clinics, we identified all patients (n=128) who had switched from sublingual or peroral OAT, to LAI buprenorphine during the first two years of implementation (2019-2021 in three clinics, 2021–2023 in the fourth). In this within-subject mirror study, patients served as their own controls. The number of days of inpatient care and ER visits were extracted from the electronic medical records using a standard operating procedure. Quasi-Poisson models were used to compare health care utilization before and after starting LAI buprenorphine.

**Results:**

Among the 128 patients, 28 (22%) were female and mean age was 37.8 (SD 9.0) years. Days of inpatient care were reduced by half, dropping from an average of 9.0 to 4.5 days (incidence rate ratio [IRR]: 0.5, 95% CI 0.41–0.61, p<.001), with a health care cost reduction of SEK 45,081 (~USD 4,090) per patient. The decline in days of inpatient care was even steeper among the 85 patients who remained on LAI buprenorphine treatment at 12 months (IRR 0.26, 95% CI 0.20–0.34, p<.001), with a decrease in health care costs of SEK 73,210 (~USD 6,642) per patient. ER visits showed no significant change (IRR: 0.93, 95%CI 0.79 - 1.09). One-year treatment retention to LAI buprenorphine was high (67%). Most patients who discontinued LAI buprenorphine transitioned to other sublingual/peroral forms of OAT, resulting in an overall OAT continuation rate of 89%.

**Conclusions:**

Implementation of LAI buprenorphine in OAT clinical settings was associated with a significant reduction in inpatient care and substantially reduced health care costs. These results challenge common economic hesitations and support wider integration of LAI options in opioid use disorder treatment.

**Clinical trial number:**

Not applicable.

## Background

Opioid use disorder (OUD) is a major public health challenge worldwide, contributing to elevated morbidity and mortality [1]. Opioid agonist treatment (OAT) with buprenorphine or methadone alleviates withdrawal and craving, significantly reducing the risk of relapse, decreasing morbidity [1–4], and cutting opioid-related mortality by half [5].

According to national regulations [6, 7], OAT in Sweden must be provided by medical staff at specialized psychiatric or addiction clinics, registered with the Health and Social Care Inspectorate (IVO) [6]. All treatment is publicly funded and provided either in public regional health care centers or publicly funded private specialised clinics.

Studies suggest high retention rates in Swedish OAT programmes, with one-year retention rates of 89% [8] for methadone and 75% for buprenorphine maintenance treatment [9]. However, wider knowledge of retention in clinical settings at a national level is lacking and opioid-related mortality remains a major concern. There is no current national register for OAT, therefore the number of patients in OAT is unknown. It has been estimated to approximately 4000 patients in OAT in 2017 [10] and around 7500 in 2022 [11]. Access to OAT is uneven and lower in Sweden compared to other Nordic countries [11, 12], due to regulatory and financial barriers. Swedish national guidelines, indicate buprenorphine-naloxone as first-line treatment and methadone as the alternative, with a much lower priority for mono-buprenorphine, due to the risk for diversion. Guidelines, last revised early 2019, do not include long-acting injectable (LAI) buprenorphine formulations as they were approved after the revision in 2019 [7]. One key obstacle to OAT access is the requirement for daily supervised dosing during the first three months of treatment, and significantly longer in patients with complex needs and continued substance use [13]. While aimed at preventing diversion and misuse, this policy can create unnecessary friction for both patients and providers, limiting engagement and long-term treatment retention [14].

LAI buprenorphine formulations, administered as weekly or monthly subcutaneous injections, offer a promising alternative by reducing the need for daily clinic visits while maintaining therapeutic efficacy and reducing the risk for diversion [15, 16]. Patient-reported outcomes also suggest that LAI formulations are associated with greater convenience, reduced stigma, and improved treatment satisfaction compared to sublingual options [17–19]. Treatment with LAI buprenorphine was approved in 2019 and 2020 respectively in Sweden for the two available formulations (Buvidal® and Sublocade®, approved as Subutex ® depot in Sweden).

Nevertheless, the implementation of LAI buprenorphine has been cautious, in part due to concerns about higher medication costs and in part due to potential unintended consequences such as reduced clinical contact, support and compromised therapeutic relationship potentially leading to increased instability and health care utilization [20]. Economic evaluations have yielded mixed results: while some studies suggest cost savings from reduced clinic visits and improved adherence [21, 22], others argue that LAI formulations may not be cost-effective in the whole OUD population, though potentially beneficial for those with severe or complex clinical conditions [23, 24]. To date, few studies have investigated the impact of LAI buprenorphine on health care utilization and costs from a broader perspective, including both inpatient admissions and emergency room (ER) visits, and over longer follow-up periods. One prior study suggested a short-term reduction in ER visits after LAI initiation but was limited to six-months [25].

To address this gap, our primary aim was to examine changes in health care utilization (days of inpatient care and number of ER visits), using data from medical records from four Swedish OAT clinics, the year before and after patients transitioned from sublingual/peroral OAT medications to LAI buprenorphine. A secondary aim was to investigate treatment retention the first year following the switch to LAI buprenorphine. We aimed to evaluate whether concerns about raised health care costs and increased instability following implementation of LAI buprenorphine are justified.

## Methods

### Study design and population

The current study is part of a broader evaluation of the clinical introduction of LAI buprenorphine in OAT in Sweden [18, 20, 26]. The study was approved by the Swedish Ethical Review Authority, nr 2020–00796.

The inclusion criteria were having switched from sublingual buprenorphine or methadone to LAI buprenorphine and having received at least one LAI buprenorphine injection during the first two years after implementation of LAI buprenorphine in Sweden, at one of the four different OAT clinics. In three of the clinics, LAI buprenorphine was introduced in February 2019 immediately following the national approval. In these clinics follow-up time was February 2019 to February 2021. The fourth clinic adopted LAI buprenorphine two years later due to financial constraints, and thus the two-year observation period was February 2021- February 2023. No exclusion criteria were applied, allowing for a comprehensive and inclusive sample.

We used electronic medical records (eMRs) to identify all patients receiving treatment with LAI buprenorphine (*n* = 170) across the four OAT clinics. From these eligible participants, we identified patients (*n* = 128) who had switched from ongoing OAT with sublingual buprenorphine. The remaining 42 had started OAT with LAI buprenorphine, without prior sublingual or peroral OAT experience. These 42 patients were not included in further analyses, since this would bias analyses of health care consumption, owing to the significant decrease in morbidity and mortality when in OAT, compared to no treatment.

For each patient, data was collected for 1 year before LAI buprenorphine initiation (baseline), and at 1, 3, 6 and 12 months after the first LAI buprenorphine dose. This within-subject design enabled each patient to serve as their own control, facilitating direct comparisons before and after the transition to LAI buprenorphine.

The four OAT clinics were situated in different areas of Sweden and encompassed both urban and rural populations. All clinics were part of the tax funded national health care system with general access to OAT and subject to the same regulations and national guidelines. The clinics differed regarding clinical routines and priority setting. One clinic (clinic 3, *n* = 49) with a stronger harm-reduction focus, prioritized only patients with severe OUD, multiple comorbidities, ongoing illicit substance use and often precarious socioeconomic situations to LAI treatment. The other three clinics catered for broader OAT populations offering LAI buprenorphine to patients in different treatment phases.

### Data collection and variables

Data from the eMRs was extracted according to a predefined standard operating procedure, according to the variable definitions below, by four psychiatry residents and one specialist in psychiatry.

Demographic variables and psychiatric and medical co-morbidity diagnoses (anxiety, depression, ADHD, PTSD, bipolar disorder, psychotic disorder, autism spectrum disorder, hepatitis C) were collected at baseline or the year prior to baseline. *Alcohol and/or substance use disorder (AUD/SUD)* was defined as yes, if any addictive disorder other than OUD was set in the eMR at baseline or prior to baseline.

*Prior OAT:* Information on prior OAT was collected at baseline; type of OAT (mono-buprenorphine, buprenorphine-naloxone, methadone) and dose. Many of the patients had been in OAT for many years. Collection of detailed data on time in OAT, was not always possible, due to lack of access to data outside the scope of the study (one year prior and one year after start of LAI buprenorphine), due to patients relocating between units, as well as due to changes in eMR systems used in the different Regions.

*Treatment phase*: was based on information in the medical records on substance use and socioeconomic situation, and on the judgement of the physician collecting the data. Data on treatment phase was collected at baseline and at 12 month follow up and was coded as active use (active drug use, either self-reported by the patient or verified with drug screens), early remission (3–12 months of no drug use, or possibly minor relapse, more stable socioeconomic situation) or sustained remission ( > 12 months with no substance use, where physicians collecting the data considered, based on text in the medical records, the patient to be stable with a stable socioeconomic situation).

*Retention to treatment:* patients who discontinued with LAI buprenorphine could remain in OAT, transitioning to sublingual buprenorphine or methadone. We therefore collected data on retention to LAI buprenorphine specifically (Buvidal® and Sublocade®) as well as sublingual/peroral OAT (sublingual mono-buprenorphine, buprenorphine/naloxone, methadone) and overall OAT continuation rate (including both LAI buprenorphine and sublingual/oral formulations).

*Type of LAI buprenorphine* or if discontinued *type of sublingual/peroral OAT medication*, when applicable: were collected at 1, 3, 6 and 12 months after initiation of LAI buprenorphine. Weekly or monthly Buvidal® injections were most common. Sublocade® was approved in 2020 in Sweden, but was for a few years, during the pandemic, not available in Europe. A few clinics holding a special permit from the Swedish Medical Products Agency, had access to Sublocade® even during the study period. *LAI buprenorphine dosing:* to enable comparison of doses, we converted all monthly Buvidal® doses to weekly dose equivalents, according to the product label. Since doses and half-life are different, patients treated with Sublocade® were not included in analyses regarding medication dose.

*Illicit substance use:* was coded yes based on positive drug screens and/or self-reported intake recorded by the staff in the eMR and was collected at 1, 3, 6 and 12 months after LAI buprenorphine initiation.

*Health care utilization:* the number of days of medical and psychiatric inpatient care, as well as the number of both general and psychiatric ER visits, were collected from the eMRs from one year before and one year after the first LAI buprenorphine dose. For health care costs, we used standardized costs per visit and per days of medical and psychiatric inpatient care provided by one of the regional health care providers (Region of Östergötland).

### Statistical analysis

Data was tested for normality and homogeneity of variance. For descriptive statistics, owing to skewed data and unequal group sizes, continuous variables were evaluated using Kruskal-Wallis rank sum test. Categorical variables were evaluated with Chi-square test, and Fisher’s exact test, when applicable. Missing values (owing to missing data or loss to follow-up) were excluded from the analysis.

Data on health care utilization (number of days of inpatient care and number of ER visits) were skewed, approximating a Poisson distribution. For comparison between health care consumption before and after LAI buprenorphine initiation, both parametric (paired t-test) and non-parametric tests (Wilcoxon signed-rank test) were employed, as utilizing both methods also allowed for detection of potential influence from outliers. We then applied mixed effects Quasi-Poisson models, controlling for ADHD, AUD/SUD and clinic, to compare health care utilization, measured with days of inpatient care and ER visits, one year before and one year after the initiation of LAI buprenorphine in patients who had transitioned from sublingual or peroral OAT, to LAI buprenorphine. Sensitivity analyses were performed where we included only patients who remained on LAI buprenorphine at 12 months (*n* = 85).

Statistical analyses were performed in RStudio (Version 4.3.2) and SPSS (version 29).

## Results

Sample characteristics of the study population are presented in Table 1. Among the 128 patients, 28 (22%) were female and mean age was 37.8 (SD = 9.0) years. Prior OAT medication type differed significantly between clinics, especially regarding the use of mono-buprenorphine. In one clinic 96% of all patients were on mono-buprenorphine prior to switching to LAI buprenorphine, while in another clinic none, denoting differences in clinical practice. Psychiatric comorbidity was in general very common, with most patients (73%) having at least one comorbid psychiatric disorder, and one third (33%) had in addition to OUD a comorbid addictive disorder (AUD/SUD). Prevalence of comorbid psychiatric disorders also differed significantly between clinics. In the whole sample, one third had at least one comorbid AUD/SUD, but prevalence varied between 4 and 61% between clinics. Similarly, 43% of the sample had ADHD but prevalence varied significantly between clinics.

**Table 1:** Sample characteristics

	Total population	Clinic 1	Clinic 2	Clinic 3	Clinic 4	
	N = 128	N = 40	N = 24	N = 49	N = 15	*p*^*1*^
Female, n (%)	28 (22)	8 (29)	6 (25)	19 (20)	4 (27)	0.9
Age, mean (SD)	37.8 (9.0)	35 (9.1)	37 (11.0)	41 (8.1)	36.7 (5.6)	0.005
Prior OAT^2^, n (%)		40 (73)	24 (77)	49 (93)	15 (48)	
Buprenorphine/naloxone,	61 (48)	36 (90)	11 (46)	1 (2)	13 (87)	< 0.001
Mono-buprenorphine	60 (47)	3 (8)	10 (42)	47 (96)	0 (0)	< 0.001
Methadone	7 (5)	1 (3)	3 (13)	1 (2)	2 (13)	0.12
Prior buprenorphine dose^3^, mean (SD)	17.0 (4.4)	16.3 (4.5)	15.0 (2.0)	18.3 (3.3)	18.0 (8.1)	< 0.001
Co-morbidity^4^, n (%)						
AUD/SUD	43 (33)	8 (20)	1 (4)	30 (61)	4 (27)	< 0.001
ADHD	55 (43)	22 (55)	12 (50)	20 (41)	1 (7)	0.01
Anxiety	43 (35)	6 (17)	3 (13)	28 (57)	6 (40)	< 0.001
Depression	21 (17)	4 (11)	3 (13)	14 (11)	0 (0)	0.03
Hepatitis C	30 (23)	7 (18)	9 (38)	10 (20)	4 (27)	0.29
Housing, n(%)						
Homeless	6 (5)	4 (10)	0 (0)	1 (2)	1 (7)	0.2
Housing via social services	29 (23)	7 (18)	9 (38)	11 (22)	2 (13)	0.22
Own housing	75 (59)	23 (58)	14 (58)	32 (65)	6 (40)	0.38
Other	18 (14)	6 (15)	1 (4)	5 (10)	6 (40)	0.01
Education, n (%)						
Elementary	59 (46)	17 (43)	13 (54)	27 (45)	7 (47)	0.83
High school	49 (38)	10 (25)	9 (38)	26 (53)	4 (27)	0.04
University	5 (4)	1 (3)	2 (8)	1 (2)	1 (7)	0.53
Treatment phase^5^, Baseline n(%)						
Active use	65 (51)	21 (53)	9 (38)	28 (57)	7 (47)	0.45
Early remission	42 (33)	18 (45)	13 (54)	9 (18)	2 (13)	0.002
Sustained remission	21 (16)	1 (3)	2 (8)	12 (25)	6 (40)	0.002
Treatment phase^5^, 12 months, n(%)						
Active use	54 (42)	16 (40)	5 (21)	27 (55)	6 (40)	0.047
Early remission	30 (23)	12 (30)	6 (25)	8 (16)	4 (27)	0.48
Sustained remission	32 (25)	8 (20)	6 (25)	13 (27)	5 (33)	0.77

^1^Pearson’s Chi-squared test/Fisher’s exact test, Kruskal-Wallis rank sum test

^2^OAT before start of LAI buprenorphine. Methadone: methadone or levomethadone

^3^Prior daily dose for those on buprenorphine/naloxone and mono-buprenorphine

^4^Co-morbid disorders classified by the ICD-10 at baseline. Anxiety; any anxiety disorder. Depressive; any unipolar depressive disorder. AUD/SUD; alcohol and/or any substance use disorder (other than OUD) (F1xx, including polysubstance use, excluding nicotine)

^5^Based on drug screens and self-reported illicit substance use, coded as: (1) *ongoing use*; for current illicit substance use, (2) *early remission*; for 3–12 months with no illicit substance use, with very few/short relapses and (3) *sustained remission*: for no illicit substance use > 12 months, with no or possibly only minor relapses. Factors such as, but not limited to, comorbidities, criminality, employment status, and housing were also considered

Missing values; anxiety disorders; *n* = 4, depressive disorders; *n* = 4, both clinic 1, treatment phase

Despite some differences in sample characteristics between clinics (see Table 1), the population overall reflects clinical reality for most middle sized to large OAT clinics in Sweden (11). At one year, one patient had died, and a minority had dropped out/were lost to follow-up (*n* = 14).

Prevalence of illicit opioid use was relatively stable over time, with around one in five (21%) reporting or screening positive for illicit opioid use at 1 month follow-up, and 24% at 12 months, after LAI buprenorphine initiation. Use of other illicit substances was common, with over half (52%) at one month follow-up and two thirds (66%) at 12 months either self-reported or testing positive on drug screens for illicit substance use. Use of multiple substances was common throughout the study period (data not shown).

### Retention to treatment

**Fig. 1:**
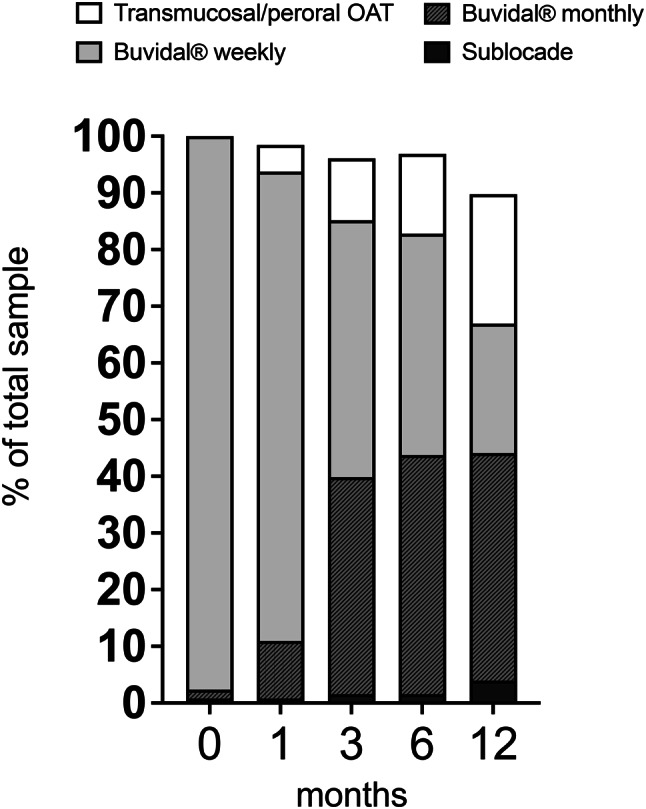
Retention to LAI buprenorphine by type (Buvidal® weekly, Buvidal® monthly, Sublocade®) or sublingual/peroral OAT (sublingual buprenorphine, methadone) at all time points for the total sample (*n* = 128). LAI buprenorphine retention; 95% at 1 month; 88% at 3 months; 82% at 6 months, and 66% at 12 months. OAT overall continuation rate; 98% at 1 month, 96% at 3 months, 97% at 6 months, and 89% at 12 months

Retention to LAI buprenorphine (Buvidal® or Sublocade®) at 12 months was 66% (Fig. 1) but varied substantially between clinics; 38% in clinic 2; 65% in clinic 1; 73% in clinic 4. In clinic 3, with a harm-reduction profile catering for patients with complex needs, 80% (*n* = 39) had ongoing LAI buprenorphine at one-year follow-up. Across the four clinics, 89% (*n* = 114) were still in OAT (LAI buprenorphine, sublingual buprenorphine or methadone) at one-year follow-up.

### LAI buprenorphine dosing

**Fig. 2:**
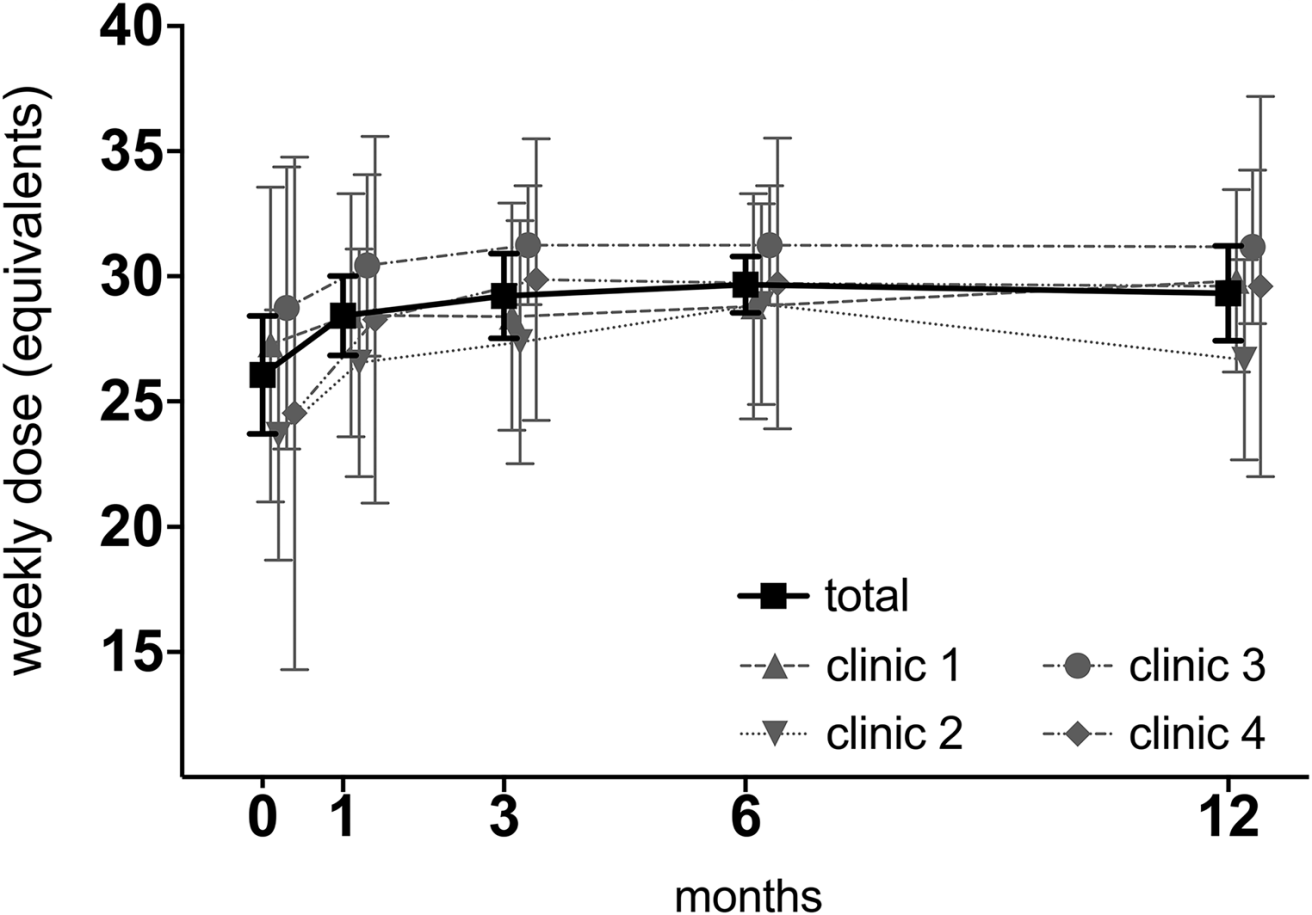
Weekly dose equivalents for participants treated with Buvidal®, for the total population (*n* = 127 at baseline, *n* = 119 at 1 month, *n* = 107 at 3 months, *n* = 104 at 6 months and *n* = 80 at 12 months) by clinic. Mean (SD) weekly dose for the total population was; 26.1 (2.4) at baseline, 28.4 (1.6) at 1 month, 29.2 (1.7) at 3 months, 29.7 (1.3) at 6 months and 29.3 (1.9) at 12 months. A weekly Buvidal® dose of 24 mg corresponds to 12–16 mg daily sublingual buprenorphine and 32 mg to 18–24 mg daily sublingual buprenorphine, according to label

Detailed dosing trajectories are illustrated in Fig. 2. Mean doses for patients on Buvidal®, expressed in weekly dose equivalents, gradually increased during the first months, reaching a plateau at around 3 months. Thereafter, dosing levels remained on a relatively stable level for the duration of the 12-month follow-up period. Across all clinics, the Buvidal® mean weekly dose equivalent exceeded 24 mg, corresponding to an approximate sublingual buprenorphine dose > 16 mg (Fig. 2). Patients on Sublocade® were excluded from this analysis (*n* = 1 at baseline and 1 month, *n* = 2 at 3 and 6 months, and *n* = 4 at 12 months).

### Health care utilization before and after initiation of LAI buprenorphine

**Fig. 3:**
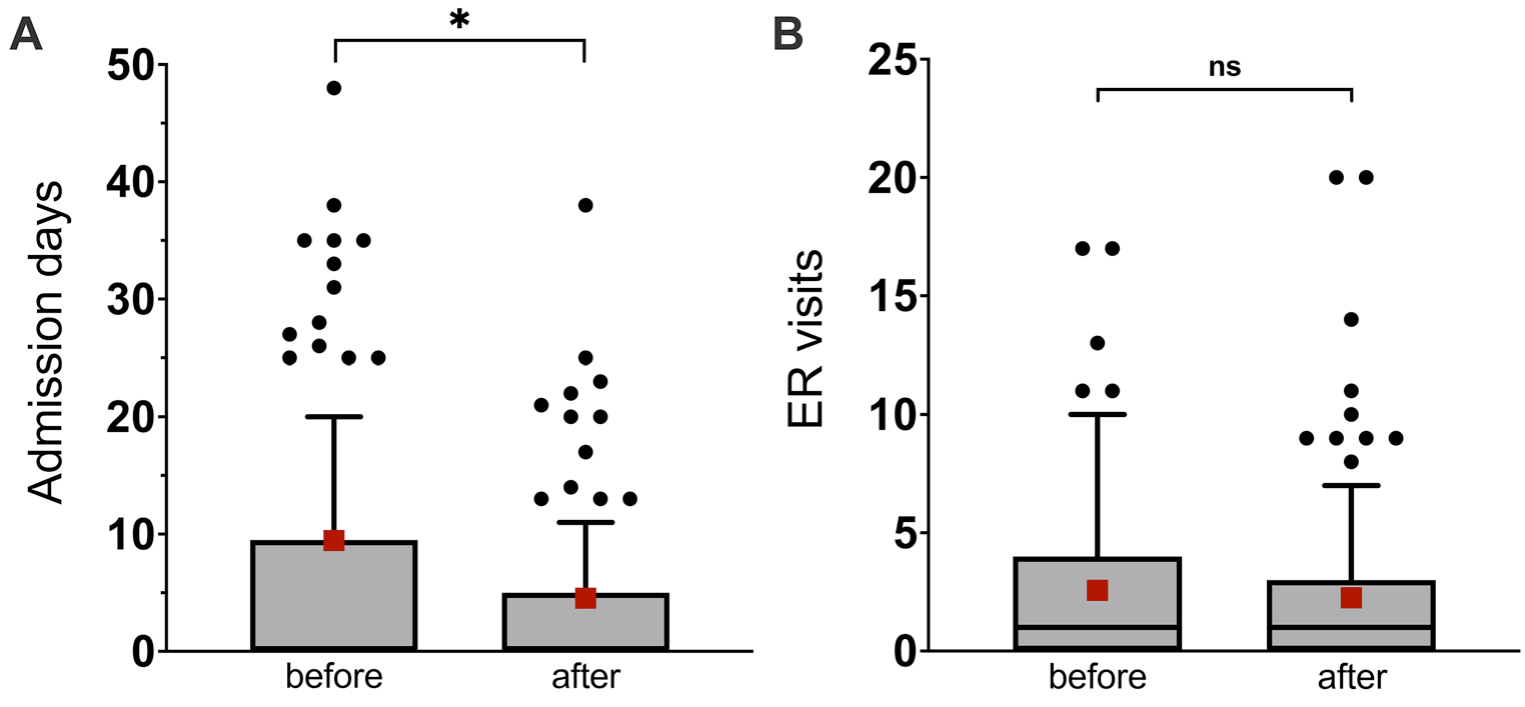
Health care utilization 12 months before and after initiation of LAI buprenorphine (*n* = 128 patients). Boxplots with Tukey method plotting whiskers and individual values, red squares denote mean values. **A**. Days of inpatient care (before: 9.0 (SD 22.2) days; after: 4.5 (SD 11.4) days, asterisk denotes p-values: t(122) = −2.23, *p* = 0.028; *p* = 0.060. Outliers, i.e. patients with > 60 days of inpatient care (*n* = 5 before, and *n* = 1 after) had been removed from the figure, to improve visualization. **B**. Number of ER visits (before: 2.45 (SD 3.16) days; after: 2.27 (SD 3.6) days) (t(122) = −0.53, *p* = 0.598; *p* = 0.370)

Changes in the number of days of inpatient care and number of ER visits the year before and the year after the start of LAI buprenorphine are presented in Fig. 3.

On average, the number of days of inpatient care decreased from 9.0 days (SD 22.2) to 4.5 days (SD 11.4) the year after start of LAI buprenorphine, compared to the year before (paired t-test and Wilcoxon signed-rank test: t(122) = −2.23, *p* = 0.028; *p* = 0.060), with a more pronounced reduction of 10.2 (SD 25.2) to 2.7 (SD 5.3) days (t(81) = 2.70, *p* = 0.009; *p* = 0.002) in those with continued LAI buprenorphine at 12 months (*n* = 85). The number of ER visits was practically unchanged (t(122) = −0.53, *p* = 0.598; *p* = 0.370).

The regression analysis rendered similar results, indicating a reduction by half (incidence rate ratio [IRR]: 0.5, 95%CI 0.41–0.61, *p* < 0.001) of days of inpatient care the year after switching to LAI buprenorphine, compared to the previous year, when controlling for clinic, and baseline ADHD and AUD/SUD. The sensitivity analysis, including participants still on LAI buprenorphine at 12 months (*n* = 85), showed a 74% reduction of days of inpatient care (IRR: 0.26, 95%CI 0.20 - 0.34, *p* < 0.001). The number of ER visits also decreased, but the effect was not significant (IRR: 0.93, 95%CI 0.79 - 1.09). The presence of comorbid addictive disorders significantly contributed to an increased risk for ER visits (Table 2).

**Table 2:** Health care utilization measured in number of days of inpatient care and number of emergency room (ER) visits, expressed in incidence rate ratios (IRR) obtained from mixed-effects quasipoisson models including co-variates

	Total population n = 128				LAI buprenorphine at 12 months (n = 85)			
	Days of inpatient care		ER-visits		Days of inpatient care		ER-visits	
	Coef (SE)	IRR (95%CI)	Coef (SE)	IRR (95%CI)	Coef (SE)	IRR (95%CI)	Coef (SE)	IRR (95%CI)
Intercept	0.345		0.408		0.577		0.451	
	(1.057)		(0.243)		(1.385)		(0.301)	
12 months	−0.694***	0.50	−0.076	0.93	−1.343***	0.26	−0.195*	0.82
	(0.101)	(0.41 - 0.61)	(0.083)	(0.79 - 1.09)	(0.139)	(0.20 - 0.34)	(0.098)	(0.68 - 1.00)
ADHD	0.222	1.25	−0.045	0.96	−0.105	0.90	0.06	1.06
	(1.032)	(0.17 - 9.43)	(0.238)	(0.60 - 1.52)	(1.368)	(0.06 - 13.15)	(0.293)	(0.60 - 1.89)
AUD/SUD	1.534	4.64	0.810**	2.25	2.143	8.52	0.997**	2.71
	(1.254)	(0.40 - 54.14)	(0.289)	(1.28 - 3.96)	(1.524)	(0.43 - 169.13)	(0.334)	(1.41 - 5.22)
Clinic 2	0.458	1.58	0.354	1.42	0.249	1.28	0.525	1.69
	(1.391)	(0.10 - 24.17)	(0.321)	(0.76 - 2.67)	(2.118)	(0.02 - 81.46)	(0.454)	(0.69 - 4.11)
Clinic 3	−2.503	0.08	−1.067***	0.34	−3.432*	0.03	−1.214***	0.30
	(1.391)	(0.01 - 1.25)	(0.313)	(0.19 - 0.64)	(1.689)	(0.00 - 0.88)	(0.357)	(0.15 - 0.60)
Clinic 4	−0.271	0.76	−0.111	0.89	−0.074	0.93	0.278	1.32
	(1.713)	(0.03 - 21.9)	(0.398)	(0.41 - 1.95)	(2.128)	(0.01 - 60.18)	(0.461)	(0.54 - 3.26)
*n*	*246/123*		*246/123*		*164/82*		*164/82*	

+ < 0.1, * < 0.05, ** < 0.01, *** < 0.001. *n* = n observations/groups. AUD/SUD: any substance and/or alcohol use disorder diagnosis, other than OUD, recorded in the medical records at baseline and the year prior to starting LAI buprenorphine

### Health care costs

Using costs provided by the tax funded, regional health care provider, we found that the annual inpatient health care costs for OAT patients after starting LAI buprenorphine was reduced almost by half, from SEK 12,334,379 to SEK 6,556,214, corresponding to a reduction of health care costs at around SEK 45,081 (~USD 4,090) per patient the first year after transitioning from sublingual to LAI buprenorphine. For the subgroup of 85 patients with continued LAI buprenorphine during the entire follow-up period, the cost reduction was 70%, from SEK 8,883,547 to SEK 2,652,088, corresponding to a decreased annual health care cost of SEK 73,210 (~USD 6,642) per patient. Costs for ER visits decreased modestly, by around 12%, corresponding to SEK 1,477 (~USD 134) per patient, this reduction however was not statistically significant.

## Discussion

In this study, we explored health care utilization before and after the introduction of LAI buprenorphine in clinical practice across four OAT clinics in Sweden. In contrast to concerns regarding the risk of increased instability with less frequent clinic visits [20], we observed a 50% decrease in the number of days of inpatient care following transition from sublingual or peroral OAT to LAI buprenorphine. An even steeper decline in number of inpatient days, was seen for patients who continued with LAI-buprenorphine at one-year follow-up. The number of ER visits did not change significantly. The reduction in days of inpatient care supports this study’s hypothesis that LAI buprenorphine is a potentially cost-effective alternative to traditional OAT medication.

Notably, LAI buprenorphine doses > 24 mg in all four clinics in this sample, corresponding to sublingual buprenorphine doses > 16 mg/d were at a level indicated by previous research as optimal and therefore recommend in guidelines [3, 27, 28], possibly contributing to the high levels of retention to treatment. Importantly, although not the focus of the study, participants who discontinued treatment with LAI buprenorphine remained in OAT. This rendered a one-year OAT retention rate of 86% on any form of OAT, which is higher compared to international research, but in line with previous Swedish data [8, 9].

Most patients in our cohort were not admitted to any inpatient care during the study period and the decrease in days of inpatient care was primarily driven by patients who previously had the highest number of inpatient care days. This trend is consistent with existing evidence that a relatively small subgroup of patients is often responsible for a substantial proportion of health care expenditures [29, 30]. Similarly, a recent study applying broad societal perspective [24] found that LAI buprenorphine was not cost-effective compared to sublingual/oral OAT, except among patients with more severe opioid related problems (as rated by the Clinical Global Impression–Severity) at baseline, or who had received traditional OAT for more than 28 days before study enrollment. This suggests that patients with high health care needs may benefit most from LAI buprenorphine treatment and extending access to this treatment option for such patients may not only offer clinical advantages but also contribute to lowering health care costs.

In a prior economic evaluation of a simulated OUD cohort conducted by Flam-Ross et al. [23], including a wide range of associated health care and treatment costs, LAI buprenorphine was not found cost-effective compared to transmucosal buprenorphine. The findings were robust to a wide range of changes in key parameters, except for substantial alterations of retention rates and medication cost. First, if the 6-month retention of LAI buprenorphine increased from 29% to 83%, LAI buprenorphine was not more costly compared to transmucosal/peroral OAT. In our cohort, 6-month retention to LAI buprenorphine was 82%, and at 12 months retention was 67%. While these retention rates are slightly lower than those observed in some cohorts [31, 32], they are significantly higher compared to those previously reported in the literature [16, 33, 34], likely related to differences in settings and follow-up time. Second, according to the same previous article, medication costs need to be decreased by 80% for LAI buprenorphine to be cost-effective [23] compared to traditional OAT. Moreover, two other studies reported monthly LAI buprenorphine injections to be the least costly OAT option [21, 22], one of these [22] conducted in Norway, a setting comparable to Swedish OAT programs. While medication cost analyses were outside the scope of this study, we argue that overall health care costs need to be considered when analyzing cost-effectiveness of various OAT options, including cost-reduction due to reduced need for inpatient care.

### Strengths and limitations

A notable strength of the study is that it encompasses all patients switching to LAI buprenorphine four OAT clinics, encompassing both urban and rural areas. Although retrospectively collected, data was prospectively recorded in the eMR, allowing following even the most unstable patients, who probably would have been excluded in randomized trials, or dropped out of prospective studies. Using eMR permitted follow-up of the whole sample with very low attrition.

Several limitations should be acknowledged. The overall sample size of 128 patients may appear modest. It is, however, comparable to or larger than many previous observational and implementation studies on long-acting injectable (LAI) buprenorphine (e.g. [25, 31]). Importantly, the study offers detailed, patient-level data across multiple time points, which is rarely available in larger registry-based studies. Additionally, the inclusion of patients from four clinics with varying clinical routines enhances the external validity of the findings, capturing a broad spectrum of real-world practice. This heterogeneity mirrors the diversity of clinical practice and strengthens the external validity of the findings. By including both urban and rural clinics with varying implementation strategies—from broad inclusion to prioritizing patients with complex needs, the study provides a representative view of LAI buprenorphine use in a real-world setting. Baseline differences across clinics should however be considered when interpreting the results. Due to the lack of access to medical records from other regions, there is a potential risk of missing data and incomplete data coverage regarding days of inpatient care and ER visits. However, this risk is deemed to be minor and unlikely to significantly affect the results, given that loss to follow up was small and most patients continued their treatment within the same region. Also, we did not collect diagnoses for admissions or ER visits. However, regarding clinical stability, previous research has shown a decrease of all-cause morbidity and mortality, and in this smaller population we would not have had the power to detect and analyze subgroups. To address the issue with regression to the mean (RTM), inherent in single-group pre–post designs, we applied a within-subject mirror design with symmetric 12-month observation windows and adjusted for baseline comorbidities and clinic differences. Health care costs were based on standardized costs from one of the health care providers (Region Östergotland). There may be small variances in costs between clinics, but since Sweden has a taxpayer funded national health care system these differences are expected to be small. Finally, prior OAT was defined as current OAT when transitioning to LAI buprenorphine but time in OAT was not registered. This limits the ability to fully assess the influence of treatment history on subsequent outcomes. Studies with larger populations and more comprehensive longitudinal data, also combining available regional and national registers, are needed to fully explore the impact of LAI buprenorphine implementation over time.

## Conclusions

This study, based on clinical implementation of LAI buprenorphine in Sweden, demonstrates a significant reduction in inpatient care following the transition from sublingual to LAI treatment even among patients with high clinical needs. We found no evidence of increased clinical instability after switching to LAI buprenorphine. In combination with substantial reductions in inpatient health care costs, these findings challenge the validity of economic arguments against broader implementation of LAI buprenorphine especially in patients with complex clinical needs. The results strongly support LAI buprenorphine as a safe, effective, and potentially cost-saving option within routine OUD care.

## Data Availability

Raw data for this project are not publicly available to preserve individuals’ privacy under the European General Data Protection Regulation and in accordance with project approval from the Swedish Ethics Review Authority.

## Abbreviations

ADHD: Attention deficit hyperactivity disorder
AUD/SUD: Alcohol and/or substance use disorder
ER: Emergency room
eMRs: Electronic medical records
LAI (buprenorphine): Long-acting injectable (buprenorphine)
IRR: Incidence rate ratio
OAT: Opioid agonist therapy
OUD: Opioid use disorder
PTSD: Post traumatic stress disorder
SEK: Swedish crowns
USD: US dollars
